# Repellent and Lethal Activities of Extracts From Fruits of Chinaberry (*Melia azedarach* L., Meliaceae) Against *Triatoma infestans*

**DOI:** 10.3389/fvets.2018.00158

**Published:** 2018-07-26

**Authors:** Martín Dadé, Pedro Zeinsteger, Facundo Bozzolo, Nora Mestorino

**Affiliations:** Laboratory of Pharmacological and Toxicological Studies (LEFyT), Faculty of Veterinary Science, Universidad Nacional de La Plata, La Plata, Argentina

**Keywords:** *Melia azedarach*, extracts, *Triatoma infestans*, repellent activities, lethal activities

## Abstract

*Triatoma infestans* is the principal vector of *Trypanosoma cruzi*, parasite responsible of Chagas's Disease transmission in Argentina. Pyrethroids have become common pesticides for the control of *T. infestans* but increasing resistance encourages the search of new alternatives and the use of natural products for biological control arises as a new strategy. *Melia azedarach* L. is originated from the Himalaya's region and several compounds are part of its rich phytochemistry. Folk medicine of the plant is due to its repellent and insecticidal activities. Aims of this work were to evaluate the repellent activity of methanolic and acetonic extracts from fruits of *M. azedarach* by means of the area preference method of fifth and first nymph stages as well as to test the acute lethal effect of the more repellent extract by means of direct application on cuticle on both stages. For repellence, qualitative filter papers were divided into two halves, one treated with methanolic (ME) or acetonic (AC) extract and the other without treatment. Controls were impregnated half with methanol or acetone and half without the solvents. One nymph was located in each Petri or well and repellence percentage was determined. For the lethal effect, fasted and fed to repletion 5th stage nymphs were topically administered with different concentrations of AC and deaths were registered after 24, 48, 72, 96, and 120 h. Phytochemical analysis of extracts was performed as well. AC demonstrated high repellent activity (100%, both stages), whereas ME extract activity was slight (10–21%). AC extract was selected for lethal assays due to early repellent activity. Fed to repletion nymphs were more sensitive to the lethal activity of the extract when compared to fasted nymphs (LD_50_: 11.5 vs. 23.1 μg/insect, respectively). Phytochemistry assays of extracts showed a higher concentration of flavonoids, alkaloids and triterpenes for AC. Considering these results, next assays will include the test of *Melia azedarach* extract on *T. infestans* that are resistant to pyrethroids for a possible synergism between AC and the pesticides.

## Introduction

Chagas's Disease, also known as American Trypanosomiasis, is a zoonosis caused by the protozoan parasite *Trypanosoma cruzi* which needs a host body and a vector to complete its life cycle, being the latter the hematophagous insect *Triatoma infestans* (“kissing bug”) distributed from Southern United States (1) to Argentina (2). The disease is endemic to Latin America and has been reported from Southern Argentina to Northern Mexico. It has also been diagnosed in people from non-endemic countries because of the increment of international migration during recent years (USA and countries from Europe, Asia and Oceania). According to the World Health Organization, up to 10 million humans are infected worldwide with more than 10,000 deaths in 2008 (3).

Transmission of *T. cruzi* is not only due to *T. infestans* hematophagous activity but also a consequence of the ingestion of contaminated food with vector stools (4), congenital infection (5, 6), blood transfusion (7, 8) and organ transplantation (9, 10). Triatomines live in dark and warm cracks of poorly-constructed homes in both rural and suburban areas. They become active during the night when they feed on hot blooded species including man. An insect usually bites an exposed area of the skin and defecates while feeding close to the bite, this situation enhances infection as the bitten person smears the feces into the bite or into the eyes, mouth or any skin lesion (3).

The evolution of the disease is characterized by two phases: acute, which may last 2 months and can be asymptomatic or with symptoms appearing shortly after the infection, they include fever, headache, enlarged lymph nodes, pallor, muscle pain, labored breathing, swelling and abdominal or chest pain (3); and a chronic phase which may last for the entire life with symptoms appearing after a silent period which may take several years. During the chronic phase, up to 30% of infected people develop lesions that compromise the heart, and up to 10% develop digestive, neurological or mixed alterations (3). Two medications are commonly used for the treatment of the acute phase of trypanosomiasis, including nifurtimox and benznidazole, with 75–100% healing with prompt administration, particularly in cases of congenital infection (11). Both medications are effective during the acute phase but not in the chronic phase and this is one of the reasons why many strategies are developed to avoid vector's transmission. Despite these pharmacological alternatives, therapeutic management of the disease is complex as adverse effects may develop during the treatment.

Many alternatives have been implemented for the interruption of disease spreading including early detection of seropositive patients and pharmacological treatment during the acute phase to avoid irreversible lesions in target organs, health campaigns and vectors surveillance by means of synthetic pesticides (e.g., pyrethroids) with residual properties (12). Pesticides are extensively used in many countries of Latin America with strong impact on non-target insects, animals and human health as well. Besides these issues, resistance to pesticide develops fast in some species of insects (13, 14) including *T. infestans* (15). Moreover, the exposition of pyrethroids to sunlight and water determines a substantial reduction of residual power (16), a common situation in rural areas. To minimize these factors new technologies and management strategies are necessary to obtain less hazardous and more resistant chemical or biological compounds. Regarding the latter, the use of plant extracts arises as a promising alternative nowadays, something that it is not necessarily new because botanical insecticides have been used for at least 2000 years in Asia and Middle East (17). Interest in these compounds relies on their low cost, efficacy, degradability and pharmacological activity on insects (18, 19).

*Melia azedarach* L. (MA) also known as “chinaberry tree” is an ornamental species of the Meliaceae family considered to be native from Asia which grows from North to South America as well as Northern Australia, Africa and Southern Europe (20). It is a deciduous and evergreen 3–10 m tree with sweet-scented lilac flowers during autumn and spring, dark green leaves and a round-shaped fruit initially green and yellowish when mature (21). Trees are cultivated in countries with template to warm climates and in Argentina they are easily found in houses and parks as ornamental trees for protection against sunlight and winds.

*Melia azedarach* has demonstrated to have both pharmacological and toxicological properties. Fruits have been studied for their phytochemical composition which includes melianoninol, melianol, melianone, meliandiol, vanillin, and vanillic acid (22). Toxic compounds are tetranortriterpens, known as meliatoxins, present in all the parts of the plants but specially concentrated in the ripe fruits (21). Aqueous and alcohol extracts prepared from different parts of MA have antibacterial (23), antiparasitic (24), antifungal (25), antiviral (26), and antioxidant properties (27) while the ingestion of foliage or fruit by cattle (28), pigs (29), dogs (30), and other species has caused intoxication with fatal outcomes in some cases. MA has also been tested for insecticide activity. Vergara et al. (31) and Carpinella et al. (25) state that this capability is due to the anti-feeding effect of tri-terpenoids that inhibit food intake capacity, which has been demonstrated in phytophagous insects, thus leading to death and malformations in next generations. Plant extracts prepared from leaves or fruits have been tested on bean weevil (32), mosquitoes (33, 34) and moths (35). Some information exists regarding repellent and insecticidal properties on *T. infestans* (36) but no up-to-date data are available.

Purpose of this work is to present the results of tests performed with acetonic and methanolic extracts prepared from fruits of MA considering repellent and insecticidal capabilities on different evolutionary stages of *T. infestans* for a possible economic and easy to implement complement of traditional pesticides used for the control of the vector of Chagas disease.

## Materials and methods

### Plant material

Ripe fruits of MA (1 kg) were collected in August 2017 from trees located close to the School of Veterinary Science, National University of La Plata (UNLP). Voucher specimens were deposited after botanical identification at the Laboratory of Pharmacological and Toxicological Studies (“LEFyT,” from Spanish), School of Veterinary Sciences, UNLP. Plant material was put inside an Erlenmeyer (2 L) and was shaken, after this 10 g of fruits were separated for the assays. Fruits were washed with distilled water and excess of moisture was removed on adsorbent paper. Covers were separated from the seeds for a better extraction and all the plant material was placed in a Soxhlet cartridge.

### Melia azedarach extracts

For the extraction, acetone or methanol (200 mL) was used as solvent for the preparation of the acetonic extract and methanolic extract, respectively. Ten grams of fruits were used to obtain each extract. The extraction temperature of the Soxhlet equipment ranged from 60 to 70°C and the process was carried out during 10 h under dim light to avoid possible inactivation of photosensitive compounds. The acetonic (AC) or methanolic (ME) extract was separated into two parts, 50 mL for phytochemical assay and 150 mL for biological assay in triatomines. The solvent of the 150 mL fraction (AC or ME extracts) was rotaevaporated at 60°C (Senko Ltd.) and a dark red residue was obtained which was resuspended in the same solvent (50 mL). This process was carried out as three consecutive extractions using 10 g of *M. azedarach* fruits each in order to standardize the amount of residue in the rotaevaporator flask and for each *in vivo* assay, obtaining in average 342 ± 50 mg/50 mL AC extract and 2,524 ± 150 mg/50 mL ME extract. These stock solutions were used to prepare a series of dilutions (1:1, 1:5, and 1:10).

Qualitative chemical determinations were performed with the 50 mL of AC and ME extracts to determine the presence of compounds with potential repellent and insecticide activities. AC and ME total extracts were fractioned in three parts and chemical reactions were used for each fraction as follows: Fraction A: Shinoda for flavonoids, FeCl_3_ for tannins and phenolic OH, Iodine for lipids, Phenol 5% + concentrated H_2_SO_4_ for carbohydrates; fraction B: Liebermann-Burchard for steroids, Bornträger for antraquinones; fraction C: Dragendorff for alkaloids, Kedde for cardenolides and Rosenheim for leucoanthocyanins (37, 38). Only qualitative analysis of the extracts was performed to guarantee the presence of compounds with repellence and lethal activities (considering previous works by other researchers). Quantitative determinations considering chromatographic techniques will be part of future assays in order to determine exact ingredients of AC extract of *Melia azedarach* in our laboratory.

### Experimental insects

Nymphs of 1st and 5th stages of *T. infestans* susceptible to pyrethroids were from an insect colony grown at LEFyT-UNLP insectary. This colony was originated from triatomines provided by the Centro de Referencia de Vectores (CeReVe), Santa María de Punilla, Córdoba, Argentina. All the insects were free of *T. cruzi* infection.

Colonies were fed with chicken blood once a week, kept at 26 ± 2°C, 60–70% relative humidity and a light cycle of 12:12.

### Repellent activity of AC and ME extracts on 1st and 5th *T. infestans* stages

**Figure 1:**
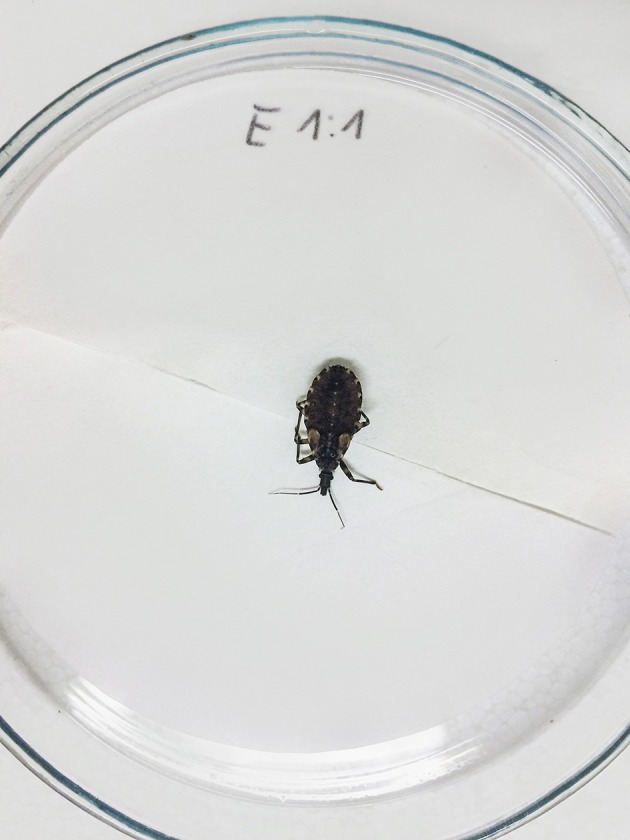
Fifth stage nymphs located into Petri dishes.

**Figure 2:**
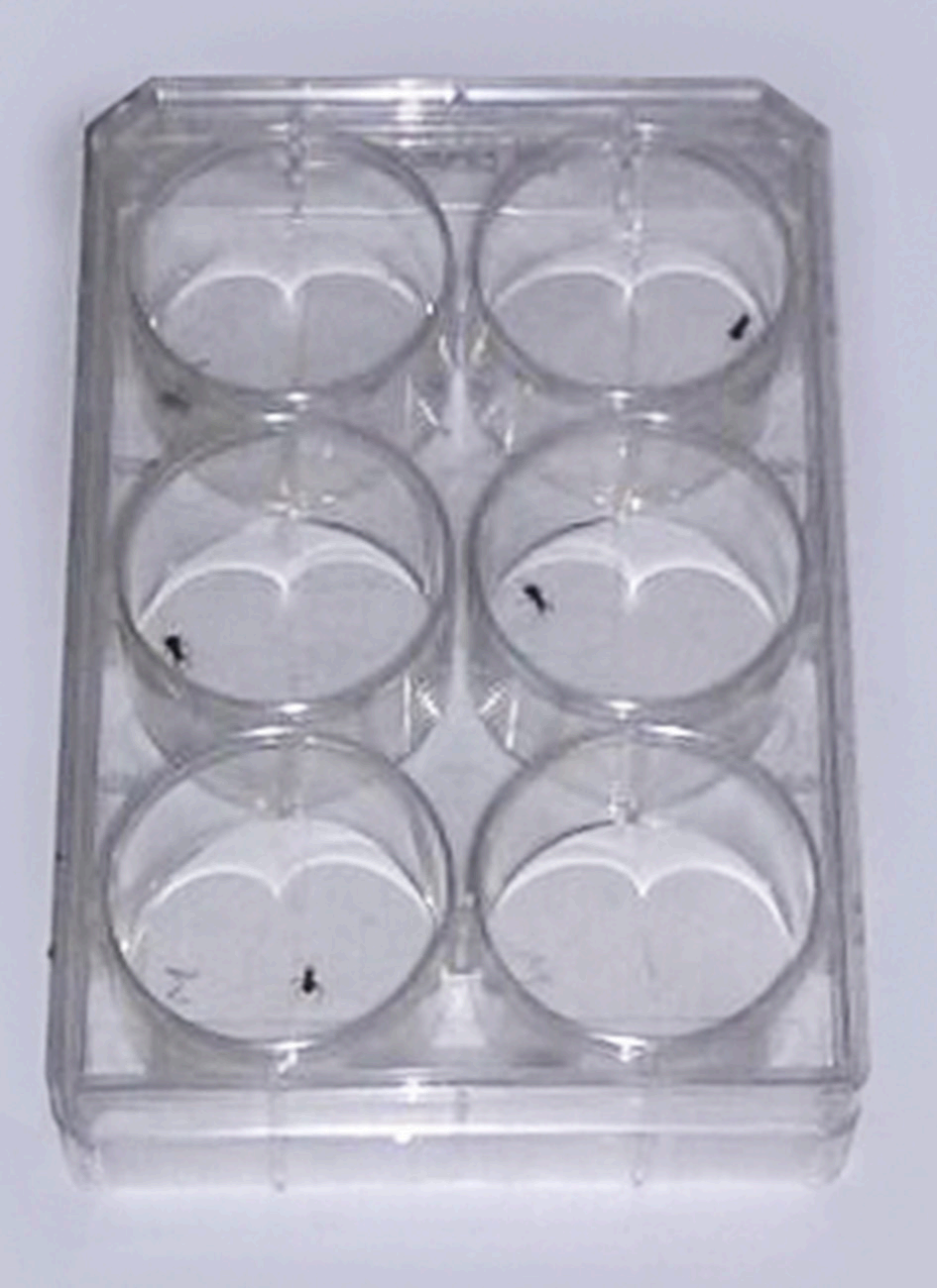
First stage nymphs located in multiple-well plates (6 wells).

For the evaluation of the repellent activity of AC and ME extracts the preference area method was used. Fifth stage nymphs were placed on a 9 cm diameter filter paper divided into two areas and then into Petri dishes (Figure 1). For 1st stage nymphs 3.5 cm filter papers were located in multiple-well plates (6 wells, Figure 2), purpose of this was to offer the insect the half of the contact surface treated with different concentrations of extract and the remaining without treatment. In case of positive repellent activity, the insect moves to the area of the paper free of extract. For controls, one half of the paper was treated with acetone o methanol. Treated halves were left to evaporate during 24 h before placement of insects and joined to the correspondent non-treated half using adhesive tape. Both nymph stages were tested using three dilutions of AC or ME extracts (1:1, 1:5, and 1:10), each concentration tested on ten insects. Volume for each dilution was 500 μL for 5th stage nymphs and 77 μL for 1st stage nymphs. Each insect was placed in the center of the paper and observation was performed after 1, 12, 24, and 48 h. Repellent activity (RA) was calculated using the following equation:

$$\begin{array}{l} RA=\frac{Nc\;-Nt}{Nc\;+Nt}\;x\;100 \end{array}$$

Nc: number of insects in the control area; Nt: number of insects in the treated area.

Values of RA may be negative or positive. In the case that most of the insects stay in the untreated area (Nc > Nt) RA value will be positive and the assayed substance is considered to have repellent activity. Negative RA values (Nt > Nc) means that most of the insects stay in the treated area, thus considering the assayed extract to have attracting activity (37).

### Insecticidal capabilities

Assays were performed only with AC extract as it showed rapid onset of repellent activity, a characteristic that was absent in ME extract. Insecticidal capability of AC extract was determined on 5th stage nymphs by means of the calculation of the LD_50_, comparing fed to repletion vs. fasted triatomines. From the stock solution of the AC extract (342 ± 50 mg/50 mL), different work solutions to be assayed were prepared by means of dilution/concentration procedures in order to obtain a concentrations range between 1.37 and 30.52 μg/μL. Briefly, the stock solution was diluted with acetone (1:5) to obtain a secondary solution (1.37 μg/μL). Afterwards aliquots were taken from the stock or secondary solution, which were concentrated to dryness (SpeedDry Vacuum Concentrators Christ CD Plus, Germany) and then dissolved in different volumes of acetone to reach the final concentrations/μL to be tested on the triatomines.

### Calculation of LD_50_ in 5th stage *T. infestans* fasted nymphs

For the determination of the LD_50_ of AC extract, 5th stage *T. infestans* susceptible to pyrethroids was used according to the WHO protocol (38). Nymphs were fasted during 12–15 days after ecdysis and prior to their use. The assay considered a binary response—dead or alive—with an independent variable (dose). AC extract was applied topically on the dorsal of nymphs' abdomen (1 μL). For the dose-response curves, increasing doses of the extract were evaluated (1.37–30.52 μg/μL or 1.37–30.52 μg/insect). The same procedure was used for controls with the application of 1 μL acetone. Assay was replicated during three different days under similar conditions and all the extracts were recently prepared for each repetition. Ten nymphs were used for each dose and repetition (30 insects for each dose) and only 10 nymphs as controls. After topication insects were located in labeled flasks and observed after 24, 48, 72, and 168 h. An insect was considered dead when it was not able to move from the center of the filter paper by its own or by means of stimulation with tweezers. Dead insects were removed and placed in labeled containers and observed again after 24 h to corroborate death.

### Calculation of LD_50_ in 5th stage *T. infestans* fed to repletion nymphs

For this experiment, 5th stage *T. infestans* nymphs fed to repletion with chicken blood were used in groups of 10 insects. Prior to this, nymphs were fasted for a period of 12–15 days after ecdysis. Each triatomine was individualized with different acrylic paint color marks (AD acrílico, Argentina) and weight before and after feeding with a precision scale (Denver Instruments, USA). After a feeding period of 30 min the quotient between weight after and before was calculated and only those insects with a 4-fold relation or more were used. For the determination of the LD_50_ the same procedure for fasted nymphs was used.

### Statistical analysis

To verify the relation among different doses of AC extract regarding mortality of nymphs the Probit method was used, which allows to associate mortality with a dose necessary to cause it. Mortality data for the treated nymphs were corrected taking into account the mortality of control nymphs by means of Abbott's Equation (39):

$$\begin{array}{l} Corrected\;\%\;mortality=\frac{\left(\%\;NT\;mortality-\%\;NC\;mortality\right)}{\left(100\%-\%\;NC\;mortality\right)}\;x\;100 \end{array}$$

Where:

*NT*: nymphs treated with different doses of AC extract diluted in acetone

*NC*: control nymphs, only received acetone.

From corrected mortality data of AC extract different dose-response curves were obtained using the POLO-PLUS software (LeOra Software Company, Petaluma, CA 2005). LD_50_ with its respective confidence interval 95% (CI 95%) was calculated as well. To determine differences in susceptibility between *T. infestans* nymph populations the ratio calculation between LD_50_ (RLD_50_) was calculated with confidence interval (CI) of 95%. LD50's are considered statistically different when 95% CI of RLD_50_ does not include number one (*p* < 0.05) (40).

## Results

### Phytochemistry of AC and ME extracts

Table 1 shows the results of the phytochemical analysis of the AC extract while Table 2 those for ME extract.

**Table 1:** Qualitative analysis of AC extract.

**Determination**	**Fraction A**	**Fraction B**	**Fraction C**
Shinoda	+		
FeCl_3_	+^a^		
Iodine	++		
Phenol 5% + concentrated H_2_SO_4_	+++		
Liebermann-Burchard		+^b^	+^b^
Bornträger		−	
Dragendorff			++
Kedde			−
Rosenheim			−

a *Two phenolic OH*.

b *Positive to triterpenes, negative to steroids*.

**Table 2:** Qualitative analysis of ME extract.

**Determination**	**Fraction A**	**Fraction B**	**Fraction C**
Shinoda	+		
FeCl_3_	+^a^		
Iodine	+		
Phenol 5% + concentrated H_2_SO_4_	++		
Liebermann-Burchard		+^b^	+^c^
Bornträger		−	
Dragendorff			+
Kedde			−
Rosenheim			−

a *One phenolic OH*.

b *Positive to triterpenes, negative to steroids*.

c *Positive to steroids, negative to triterpenes*.

### Repellent activities of AC and ME extracts

In Tables 3, 4 it can be observed that only AC extract showed repellent activity for both stages of *T. infestans*. Repellent activity was directly proportional to the concentration of the extract. Dilution 1:10 did not cause repellence or it was negligible in any stage. In the case of 1st nymph stage, 1:1 dilution showed weak repellent activity as early as 1 h of the initiation of the assay (Table 3), while 1:5 dilution demonstrated this activity at 24 h. Highest repellent activity (100%) was observed after 24 h and remained constant until 48 h.

**Table 3:** Repellent activity of AC extract on 1st stage *T. infestans* nymphs.

	**1 h**	**12 h**	**24 h**	**48 h**
**AC (DILUTIONS)**				
1:1	44 ± 51	77 ± 39	100 ± 0	100 ± 0
1:5	10 ± 18	21 ± 18	100 ± 0	100 ± 0
1:10	10 ± 18	0 ± 0	10 ± 18	11 ± 19
**ME (DILUTIONS)**				
1:1	11 ± 19	11 ± 19	0 ± 0	0 ± 33
1:5	ND	ND	ND	ND
1:10	ND	ND	ND	ND

*ND, Not determined*.

**Table 4:** Repellent activity of AC extract on 5th stage *T. infestans* nymphs.

	**1 h**	**12 h**	**24 h**	**48 h**
**AC (DILUTIONS)**				
1:1	21 ± 18	88 ± 19	100 ± 0	100 ± 0
1:5	10 ± 18	21 ± 18	21 ± 18	10 ± 18
1:10	0 ± 0	10 ± 18	0 ± 0	10 ± 18
**ME (DILUTIONS)**				
1:1	0 ± 0	10 ± 18	10 ± 18	0 ± 0
1:5	ND	ND	ND	ND
1:10	ND	ND	ND	ND

For the 5th stage repellent activity was lower compared to 1st stage. Again, only 1:1 dilution was effective and its activity started at 12 h after initiation of the experiment. This dilution reached 100% of repellence at 24 h and remained constant until 48 h.

### LD_50_ of AC extract

Table 5 shows the influence of nutritional state of nymphs on the lethal activity of AC extract. Fed to repletion nymphs were more sensitive to the lethal activity of the extract when compared to fasted nymphs. Both RLD_50_ were close to 2, this means that twice the dose was needed for fasted nymphs compared with fed to repletion nymphs.

**Table 5:** Relation between nymph nutritional state and lethal activity of AC extract.

	***n*^a^**	**LD_50_ (μg/i)^b^**	**Slope ± SE^d^**	**RLD_50_^e^**
		**(CI 95%)^c^**		**(CI 95%)**
Fasted	150	23.1 (19.1–28.1)	3.2 ± 0.6	
Fed to repletion	150	11.5 (8.5–14.1)	2.6 ± 0.5	2.0 (1.4–2.7)

a *Nymphs used for the experiment*.

b *LD_50_% (micrograms per insect)*.

c *Confidence Interval 95%*.

d *Standard Error*.

e *LD_50_ (fasted nymphs)/LD_50_ (fed nymphs)*.

## Discussion

Under our working conditions we found that AC extract showed efficacy in susceptible to pyrethroid nymphs regarding repellent and lethal activities, such situation was influenced by the feeding state of insects. A possible explanation of the higher lethal efficacy of AC extract in *T. infestans* nymphs fed to repletion could be associated to the ability of some insects to modify the mechanical properties of cuticle. This process, known as plasticization (41), involves modifications in the aqueous phase of cuticle due to changes in pH, leading to rupture of soft bonds among proteins and chitin microfibers. These reversible modifications allow procuticle to have a better elongation capacity. Plasticization has been largely studied in *Rhodnius proxilus* (42, 43), researchers found this process to be important during ecdysis and feeding, particularly in 5th stage nymphs when plasticization allows triatomines to considerably increase their original size. Moreover, in 5th stage nymphs of *T. infestans* it has been demonstrated that a feeding time as short as 1 min using an artificial feeding system is enough to start plasticization. During this process epicuticle folds are expanded and procuticle is more flexible, thus enhancing the penetration of different molecules from outside, such as pesticides (44). From our results it can be stated that in nymphs fed to repletion plasticization allowed an increased penetration of the active compounds present in AC extract resulting in a higher toxic response (LD_50_ fed to repletion insects < LD_50_ fasted insects). In 2nd stage *T. infestans* submitted to 14C-DDT, Fontán and Zerba (45) reported an increased penetration rate of the organochlorine in fed to repletion vs. fasted insects; this result is similar to the topication assay we performed with AC extract.

Regarding repellent activity our results were similar to those of Valladares et al. (36) although our extract was acetonic, compared to the ethanolic extract of the researchers. They also determined that the ethanolic extract did not affect the survival of triatomines when they were submitted to papers impregnated with extracts. The latter could be a possible explanation to the differences with our results, because in the case of topication each insect receives a determined volume (dose) while with the contact method the amount of extract on insects depends on the locomotive activity of each individual. Besides, there are some similarities and differences in the chemical composition of extracts. The ethanolic extract from Valladares et al. (36) had limonoids, a group of insecticidal triterpenes. In AC extract, although we did not determine the chemical identity of triterpenes, they were abundant. As a difference, the ethanolic extract did not have alkaloids, compounds that were present in AC extract and are probably responsible for the lethal activity, as these secondary metabolites are used as defense mechanism against insects and herbivores (46). Such compounds, acting as protective agents for plants, are known as allelochemics (47).

Acetone is the recommended solvent for experimental use in *T. infestans* according to the World Health Organization (38). We found that repellent activity of AC extract was slightly greater in 1st stage compared to 5th stage nymphs, this could be related to anatomical and physiological differences between both stages, such as penetration rate of substances through cuticle, presence of sensory organs specific to each stage and augmentation of metabolic activity (48). Repellent activity of ME extract was considerably low when compared to AC extract, this could be due to the higher concentration of some compounds in AC extract such as triterpenes, as previously demonstrated for similar compounds by other researchers (49, 50).

Although health campaigns have been implemented in developing countries the infected human population in Latin America is still high and there is concern about international immigration in countries where the disease is non-endemic. Synthetic insecticides are useful tools for the control of pests, but their excessive use has led to negative consequences such as toxicity against farmers, consumers and both wild and domestic animals as well as interruption of natural control and pollination, water pollution and development of resistance (50–53). Moreover, some populations of *T. infestans* have developed resistance to these pesticides (52, 54) *Melia azedarach* is present in many countries of Latin America where it is usually used in folk medicine by means of maceration of fruits and leaves to prepare extracts due to their repellent and insecticide properties against many crop pests and human disease vectors. Such conditions together with our findings could justify the use of plant preparations as an accessible complement together with the traditional use of pesticides for the control of *T. infestans* with probable synergism or potentiation of actions between molecules in susceptible nymphs.

Next step in our research will be the assay of MA extracts on *T. infestans* that are resistant to pyrethroids for a possible synergism between AC extract and the pesticides. Such situation, if successful, may allow to use less concentrations of synthetic insecticides during aspersion, which in turn may cause less impact on environment as well as human population. Part of this work has already started.

## Author contributions

MD, PZ, and NM conceived and designed the experiments. PZ performed the *Melia azedarach* Extracts. FB Managed and maintained the triatomines. All authors contributed to the redaction, revision and approved the final manuscript.

### Conflict of interest statement

The authors declare that the research was conducted in the absence of any commercial or financial relationships that could be construed as a potential conflict of interest.
